# Cambridge University Course

**Published:** 1921-08-27

**Authors:** 


### CAMBRIDGE UNIVERSITY COURSE.

The degrees of Bachelor of Medicine (M.B.) and Bachelor of Surgery (B.Ch.) at this University are only conferred after the entire course of study with examinations for both has been carried out. The B.Ch. is, therefore, in itself a complete and registrable qualification to practise, and is frequently so used. To obtain either of these degrees it is necessary to pass five years in the study of medicine after registration as a student, and to reside for three years at the University. The professional examinations are three in number. The first comprises physics, chemistry, and biology; like similar examinations elsewhere, it is divided into three parts, which may be passed separately. The second is divided into two parts—Part I. comprising human anatomy and physiology, Part II. elementary pharmacology and pharmaceutical chemistry and general pathology. The third is also divided into two parts—Part I. including surgery and midwifery, Part II. physics and pathology and pharmacology. When the third examination has been completed the student may take the degree of B.Ch. at once, but for the M.B. he must prepare and submit a thesis on some subject in medicine, surgery, or midwifery, selected by himself.

For the degree of M.D. it is necessary to have been M.B. at least three years, or else M.A. for at least four years, having passed all the examinations required for B.Ch., and to submit a thesis on some subject in medicine (not in surgery) which is a definite research or contribution to the knowledge of its subject.

There is also an advanced degree in surgery (Master of Surgery, M.Ch.), which is said to demand a distinctly higher standard of surgical knowledge and proficiency than the English F.R.C.S. Not many candidates attempt it, and fewer still attain it; practically all who possess it are surgeons on the staff of important general hospitals.

